# New Appliances and Things Medical

**Published:** 1897-12-18

**Authors:** 


### NEW APPLIANCES AND THINGS MEDICAL.

**[We shall be glad to receive, at our Office, 28 & 29, Southampton Street, Strand, London, W.C., from the manufacturers, specimens of all new preparations and appliances which may be brought out from time to time.]**

### "FRAME FOOD" JELLY.

(Frame Food Company, Lombard Road, Battersea, S.W.)

This jelly is a new and modified form of the well-known Frame Food extract. It is specially designed to meet the requirements of the nursery and convalescents. It may be taken alone or spread on bread, and is sufficiently pleasant to the palate to be regarded almost in the light of a condiment, and in our experience children take very kindly to it. The jelly is a compound of "Frame Food" extract and sugar, and containing as it does a large percentage of organic phosphates and albuminoids, it must be regarded as a food largely endowed with the elements which go to build up and repair the tissues of the body. In itself it is highly digestible, but further than this, it is calculated to assist rather than hinder the digestion of other foods taken at the same time.

### OXINE.

(29, Walbrook, London, E.C.)

Oxine is a concentrated extract of meat combined with vegetable products. It possesses the qualifications of a food as well as the refreshing and stimulating qualities of a simple meat extract. Being highly concentrated and free from a large excess of water, the tabloids of oxine are most convenient for travellers, and either in bulk or in small quantities this preparation can be conveniently stored for domestic purposes. Containing as it does a certain proportion of vegetable products, it is in this respect a distinct advance on the older forms of meat extracts and on most modern varieties which still continue in a conservative adherence to the mistaken notions of dietetics inaugurated some fifty years ago when laboratory experiments, though altogether inaccurate and insufficient, held sway over clinical methods and practice. Oxine is a step in the right direction as regards sick-room feeding, where the monotony of diluted milk and nauseous meat extractives have too long predominated.

### WOOD-WOOL WADDING AND TISSUE.

(The Sanitary Wood-Wool Company, Limited, 26, Thavie's Inn, Holborn Circus, W.C.)

Owing to its high powers of absorption, wood-wool has for long been most favourably regarded by the profession. The preparations of this firm, from the care with which they are sterilised, must be regarded as valuable additions to the requisites for aseptic surgery. For cases in which there is a very free discharge, as from tuberculous sinuses or cancer, wood-wool dressings are to be particularly recommended. But in fact both the wadding and the tissue are applicable to a very large variety of surgical cases. They are easily manipulated, the wadding being readily torn, and the tissue cut to any required shape, and thus they are very convenient in private practice where dressings may have to be prepared by the surgeon himself.

### INDIAN TEAS AND CONDENSED COFFEE.

(Terrabona Company, Limited, London.)

We have received samples of the above products from the Terrabona Company. The tea is a good example of a pleasant Indian blend, of good aroma, and free from large excess of tannin. The leaf is fairly uniform, free from any considerable quantity of *débris*. For a tea at 2s. per lb. it cannot be materially bettered for ordinary domestic use. The condensed coffee, which is combined with a certain proportion of chicory, appeals to us as a particularly pleasant and useful preparation, being absolutely ready for use. Nurses should welcome this innovation as a happy and occasional substitute for the large potations of indifferent tea with which they destroy their digestions. The Terrabona condensed coffee is sold in elegant little bottles, a cleanly substitute for the more frequently adopted can.

